# CK2 Regulation: Perspectives in 2021

**DOI:** 10.3390/biomedicines9101361

**Published:** 2021-09-30

**Authors:** Scott E. Roffey, David W. Litchfield

**Affiliations:** 1 Department of Biochemistry, Schulich School of Medicine & Dentistry, University of Western Ontario, London, ON N6A 5C1, Canada; sroffey2@uwo.ca; 2 Department of Oncology, Schulich School of Medicine & Dentistry, University of Western Ontario, London, ON N6A 5C1, Canada

**Keywords:** protein kinase CK2, casein kinase II, CSNK2, phosphorylation, post-translational modifications, signal transduction, kinase regulation

## Abstract

The protein kinase CK2 (CK2) family encompasses a small number of acidophilic serine/threonine kinases that phosphorylate substrates involved in numerous biological processes including apoptosis, cell proliferation, and the DNA damage response. CK2 has also been implicated in many human malignancies and other disorders including Alzheimer′s and Parkinson’s diseases, and COVID-19. Interestingly, no single mechanism describes how CK2 is regulated, including activation by external proteins or domains, phosphorylation, or dimerization. Furthermore, the kinase has an elongated activation loop that locks the kinase into an active conformation, leading CK2 to be labelled a constitutively active kinase. This presents an interesting paradox that remains unanswered: how can a constitutively active kinase regulate biological processes that require careful control? Here, we highlight a selection of studies where CK2 activity is regulated at the substrate level, and discuss them based on the regulatory mechanism. Overall, this review describes numerous biological processes where CK2 activity is regulated, highlighting how a constitutively active kinase can still control numerous cellular activities. It is also evident that more research is required to fully elucidate the mechanisms that regulate CK2 and what causes aberrant CK2 signaling in disease.

## 1. Introduction

Three mechanisms can generally explain how kinase activity is regulated based on how the kinase active sites are exposed. The first involves activation by external proteins or domains, where the kinase conformation changes upon binding to other molecules such as proteins or second messengers such as cyclic adenosine monophosphate (cAMP), thus exposing the active site. An example of a kinase that subscribes to this mode of regulation is cyclin-dependent kinase (CDK) 2 (CDK2). The second common regulatory mechanism of kinase activity applies to AGC-family kinases like protein kinase B (PKB/AKT). Here, phosphorylation of conserved residues in both the kinase activation segment and a conserved hydrophobic region towards the C-terminal are required to form the active conformation. The third kinase activation mode involves kinase dimerization, where activation occurs through allosteric interactions with another domain of the same kinase. An example of this involves checkpoint kinase 2 (CHK2), where dimerization enables reciprocal phosphorylation of the kinase activation loop, promoting the active conformation [1].

### 1.1. Protein Kinase CK2

#### 1.1.1. Background

Of the 518 protein kinases identified in the human genome, some that have gained considerable attention are from the protein kinase CK2 family (CK2; formerly known as casein kinase II). CK2 was first discovered in 1954 by Burnett and Kennedy as a “mitochondrial enzyme” from rat liver extracts that utilized adenosine triphosphate (ATP) to phosphorylate purified casein [2]. With the dramatic improvement in biochemical techniques since then, we now know a lot more about CK2.

The CK2 family is comprised of two catalytic subunits (⍺ and ⍺′) and the regulatory β subunit that generally assemble into tetrameric complexes with two catalytic and two regulatory subunits. Within the human genome, CK2⍺, CK2⍺′, and CK2β are encoded by the CSNK2A1, CSNK2A2, and CSNK2B genes, respectively. CK2⍺ and CK2⍺′ catalyze the transfer of the gamma-phosphate from ATP or guanosine triphosphate (GTP) and share 90% sequence identity, diverging in their C-termini [3]. Interestingly, even though CK2⍺ and CK2⍺′ are highly similar, proteomic studies analyzing protein abundance have revealed that CK2⍺ is typically the predominantly expressed catalytic subunit [4,5,6] A primary function of the regulatory CK2β subunit is to modulate substrate specificity via an acidic loop near residue 50 in its N-terminal. While CK2 is an acidophilic kinase, the CK2β acidic loop interacts with basic regions of some substrates, docking the kinase on the substrate to facilitate catalysis by bringing the phosphosite into proximity to the catalytic pocket [7]. There are three types of CK2 substrates which will be described in detail later.

CK2⍺, CK2⍺′, and CK2β are present in numerous cellular compartments, including the nucleus, cytoplasm, plasma membrane [8], mitochondria [9], smooth endoplasmic reticulum, and the Golgi apparatus [10]. Furthermore, CK2⍺ and CK2β have also been identified in the extracellular matrix [11]. While CK2 is ubiquitously expressed in cells, there is evidence of differential subunit expression in tissues. For example, both CK2⍺ and CK2β are highly abundant in the brain and endocrine tissues but more weakly expressed in the pancreas, liver, and gallbladder, suggesting that CK2 may have broader functional roles in some tissues than others [12]. Furthermore, CK2 is essential for survival as CK2⍺ and CK2β knockouts in mouse embryos are lethal [13,14]. Notably, CK2⍺′ knockouts are viable but lead to male infertility [15].

CK2 exists in different forms, both as tetrameric assemblies and as free subunits. The most common is the CK2⍺ holoenzyme, a tetrameric complex composed of two CK2⍺ subunits connected by a dimer of CK2β subunits. Although less prevalent, CK2 is also present as a tetrameric holoenzyme with two CK2⍺′ subunits or with one CK2⍺ and one CK2⍺’ subunit. CK2⍺, CK2⍺′, and CK2β monomers also exist within cells [3]. CK2 substrates are clustered into three groups depending on which molecular form of CK2 phosphorylates the serine or threonine residue found within its S/T-X-X-D/E/pS [16] consensus motif. Most CK2 substrates are class 1 substrates, which are comparably phosphorylated by both the holoenzyme and free catalytic subunits of CK2 [17]. Class 2 substrates, such as calmodulin, are only phosphorylated by catalytic CK2 monomers [18]. Finally, class 3 substrates like the eukaryotic initiation factor 2β (EIF2β) are only phosphorylated by holoenzyme-forms of CK2 as they require ‘docking’ by CK2β before catalysis can occur [7]. 

#### 1.1.2. CK2 in Disease

CK2 is implicated in numerous human diseases such as Alzheimer’s [19], Parkinson’s and Huntington’s diseases [20], diabetes [21], cardiac ischemia [22], and several malignancies including breast, lung, prostate, and ovarian cancers. Studies have shown differential expression of CK2 subunits [23] in different cancer types. Furthermore, there is a relationship between aberrant CK2 expression and decreased patient survival rates as CK2 has been implicated in promoting antitumor drug resistance through apoptosis evasion, increased DNA damage repair, and increased drug efflux [24]. Based on its role in human disease, CK2 has attracted attention as a potential therapeutic target which has led to the development of several notable CK2 inhibitors (Table 1). For example, in 2010, the novel ATP-competitive CK2 inhibitor Silmitasertib (CX-4945) emerged as the first clinical stage CK2 inhibitor [25]. Silmitasertib is currently being evaluated in clinical trials to treat multiple malignancies, including childhood medulloblastoma (NCT03904862), kidney cancer (NCT03571438), and cholangiocarcinoma (NCT02128282).

##### Okur-Chung Syndrome

In 2016, a noteworthy study that involved 4102 patients with neurodevelopmental disorders was published. While no studies to date have identified any gain-of-function mutations in any CK2 subunits, five unrelated female children in this study that presented with similar neurodegenerative symptoms, including developmental delay and intellectual disability behavioural problems, speech problems, and dysmorphic facial features had germline mutations in the CK2⍺ gene. Whole-exome sequencing revealed that of these five individuals, two had missense mutations within the activation segment of the kinase (D175G and K198R), another two with missense mutations within the ATP/GTP binding loop (R47Q, Y50S), and the fifth patient had an c.824 + 2T>C splice variant. All five of these mutations were predicted in silico to be deleterious and disease causing [32]. This disease, termed Okur-Chung Neurodevelopmental Syndrome [33], is the first disorder attributed to loss-of-function mutations in CK2.

##### COVID-19

In 2019, the novel coronavirus disease 2019 (COVID-19) pandemic caused by the SARS-CoV-2 virus quickly spread throughout the world, leading to intense effort to understand, diagnose, and treat the disease. Interestingly, phosphoproteomic analysis revealed that SARS-CoV-2 infection strongly affected CK2 as well as other p38 pathway-associated kinases. In addition to phosphorylating SARS-CoV-2 proteins, CK2⍺′ and CK2β physically interact with the viral nucleocapsid protein and co-localize at virus-induced filopodial protrusions. This interaction leads to increased phosphorylation of several proteins, including histone deacetylase 2 (HDAC2), high mobility group protein HMG-I/HMG-Y (HMGA1), non-histone chromosomal protein HMG-14 (HMGN1), signal transducer and activator of transcription 1 (STAT1), and catenin alpha-1 (CTNNA), and suggested that SARS-CoV-2 may regulate CK2-mediated cytoskeletal organization. An in vitro infection assay further probed the relationship between CK2 and SARS-CoV-2, where Vero E6 cells were pre-treated with the antiviral remdesivir, followed by SARS-CoV-2 infection. Forty-eight hours post-infection, cells were treated with 0.1-100 µM of Silmitasertib, resulting in a significantly reduced viral titer compared to a dimethyl sulfoxide (DMSO) control [34]. To build on these findings, Silmitasertib is currently being evaluated in clinical trials to treat both moderate (NCT04663737) and severe (NCT04668209) COVID-19 to determine if CK2-blockade is effective in patients.

#### 1.1.3. CK2: The Constitutively Active Kinase

It is evident that dysregulated CK2 activity is frequently detected in many human diseases, but how does this occur? To answer this question, we must first understand how CK2 is regulated.

One of the most interesting structural features of catalytic CK2 subunits involves its activation loop. Traditionally, activation loops are dynamic regions that control kinase activation; however, in CK2, this domain is 30 residues longer than what is seen in cyclin-dependent kinases, resulting in additional interactions with the N-terminal lobe of the kinase. These interactions have two effects on CK2: the first being to seemingly enhance catalytic activity as disruption of the N-terminal segment increases the Km of ATP from ~10 µM to greater than 500 µM. Second, CK2 is locked in its “active” conformation, resulting in the kinase being constitutively active [35].

Given the ubiquitous expression of CK2 in cells, it is unsurprising that the kinase has been implicated in numerous fundamental biological processes, including apoptosis, cell growth and proliferation, transcription, translation, circadian rhythm, and the DNA damage response. However, what is more paradoxical is how a constitutively active kinase can play such an essential regulatory role in these biological functions.

Careful regulation of cellular processes is essential, so mechanisms that regulate CK2 activity must exist to prevent aberrant signaling. Here, we summarize a selection of the expansive published literature that highlights specific biological pathways where CK2 is regulated, either through intrinsic (self) regulation, or through extrinsic regulation by proteins, stressors, oligonucleotides, and other molecules. Many of these examples can be categorized into one of the three modes of kinase regulation that were discussed previously.

## 2. Intrinsic Regulation

### 2.1. Post-Translational Modifications

**Figure 1:**
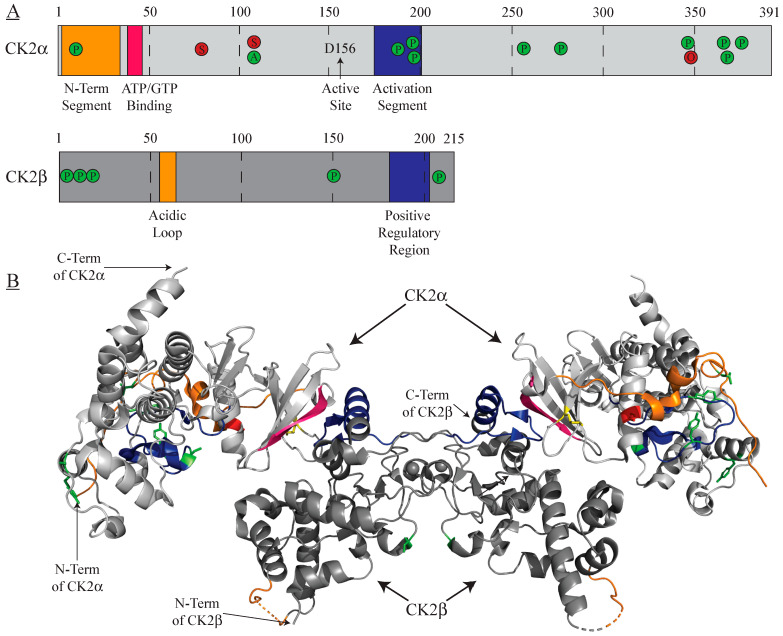
CK2⍺ and CK2β post-translational modifications. To-scale representation (**A**) and crystal structure (**B**) highlighting locations of activating (green), inhibitory (red), and both activating/inhibitory (yellow) post-translational modifications alongside selected kinase domains. A = acetylation; O = O-GlcNAc glycosylation, P = phosphorylation; S = SUMOylation. PDB: 4MD7.

CK2 can self-regulate its activity through autophosphorylation that can occur either in free catalytic subunits or within the holoenzyme. Within the free catalytic subunits, this occurs intermolecularly at Y182 on CK2⍺ and Y183 on CK2⍺′, resulting in increased catalytic activity through modulating interactions between the residue and the N-terminal of the kinase. Interestingly, holoenzyme-formation inhibits this autophosphorylation, so it can only occur to catalytic subunits not complexed with CK2β [36]. Second, autophosphorylation of the regulatory CK2β subunit occurs at serine residues 2 and 3 and potentially serine 4 when in a holoenzyme conformation [37], resulting in increased kinase activity. Given the considerable distance between the catalytic subunits and the CK2β autophosphorylation sites in the holoenzyme, this post-translational modification must also occur intermolecularly [3]. Autophosphorylation has also been implicated in protection from ubiquitination and proteasomal degradation [37] (Figure 1).

### 2.2. Protein–Protein Interactions

CK2 can also intrinsically regulate its expression and activity via protein–protein interactions. On a transcriptional level, a negative transcriptional feedback loop exists with CK2⍺. After translation of CK2⍺, it complexes with CK2β to form the holoenzyme, which can then phosphorylate its transcription factors Sp1, Ets-1, and NF-kB, resulting in decreased expression [38]. An elegant feedback loop similarly exists that controls CK2β transcription. An abundance of CK2⍺ stimulates transcription of CK2β after its transcription factors Sp1 and NF1 interact with CK2⍺ at the CK2β promoter to equalize the catalytic and regulatory subunit stoichiometries. Then, after translation of additional CK2β, it removes CK2⍺ from the promoter through holoenzyme-formation, reducing its transcription [39].

Studies have shown that CK2 also forms oligomeric superstructures that inhibit its activity. Here, CK2β initiates holoenzyme aggregation when the acidic loop on one tetramer binds to a polybasic region proximal to the catalytic substrate pocket on CK2⍺/CK2⍺′ in another tetramer, occluding the pocket and decreasing catalytic activity. Upon substrate binding to the acidic loop, the inter-tetramer interaction is released, opening the catalytic pocket and enabling phosphorylation of the substrate [40,41,42,43,44].

Overall, these studies reveal distinct complementary mechanisms by which CK2 can regulate its own activity.

## 3. Extrinsic Regulation

### 3.1. Post-Translational Modifications

#### 3.1.1. Activating

CK2 is subject to several post-translational modifications that have been associated with an increase in activity. For example, CK2⍺ has been shown to be phosphorylated by AKT at T13 within its N-terminal segment, which has been associated with enhanced ribosomal DNA (rDNA) synthesis through increased CK2-mediated phosphorylation of transcription intermediary factor 1-alpha (TIF-1⍺) [45]. Following mitogen-activated protein kinase (MAPK) pathway activation, extracellular regulated kinase 2 (ERK2) phosphorylates CK2⍺ at T360 and S362, increasing its phosphorylation of ⍺-catenin [46]. Studies have also shown that CK2⍺ is phosphorylated at Y255 by the Src-family kinases Lyn and C-Fgr, resulting in three-fold increased activity [47]. Similarly, CK2⍺ is phosphorylated at Y182 and Y188 by the Src-related kinase SRMS. However, Y188 phosphorylation causes a more pronounced increase in CK2⍺ activity [48]. During cell proliferation, increased abundance of phospholipase D2 (PLD2) stimulates protein kinase C (PKC), which activates CK2 by phosphorylating CK2⍺ at S194 and S277, and CK2β at S148 [49]. 

Interestingly, a significant number CK2 post-translational modifications occur during mitosis. First, studies have shown that CDK1 phosphorylates CK2⍺ at T344, T360, S362, and S370. This phosphorylation is strongest during prophase and metaphase before decreasing during anaphase and almost undetectable during telophase and cytokinesis. Furthermore, this phosphorylation seemingly regulates CK2 activity as phosphomimetic mutations of these residues promote cell death by mitotic catastrophe, and phosphoablative mutations cause cell cycle arrest after significant spindle damage [50]. CK2β is also phosphorylated during mitosis by CDK1 at serine 209 [51,52,53]. 

Lastly, within the transforming growth factor beta (TGFβ)-mediated activation of the epithelial-to-mesenchymal transition pathway, CK2β is phosphorylated at an unknown residue by the kinase domain of TGFβ receptor (TGFBR) 1 kinase, targeting the protein for degradation. The resulting CK2⍺:CK2β subunit abundance imbalance results in activation of CK2 [54].

While phosphorylation accounts for most CK2⍺ post-translational modifications, CK2 is also subject to other post-translational modifications including acetylation at lysine 102 that has been shown to increase its kinase activity [55,56] (Figure 1).

#### 3.1.2. Inhibitory

Post-translational modifications have also been shown to decrease CK2 activity. First, CK2⍺ S347 (which is proximal to the CDK1-phosphorylation site T344) is O-GlcNAc glycosylated. This glycosylation antagonizes CDK1-mediated phosphorylation of CK2 and increases its susceptibility to proteasomal degradation [57]. Furthermore, an analysis of SUMOylation in HEK293T cells revealed that CK2⍺ is SUMOylated. While the location of this modification is unknown, in silico SUMOplot analysis predicted that residues K79 and K102 are SUMOylated with prediction scores of above 0.75. Phosphorylation of the CK2 substrates AP1, calnexin, DNA ligase 1 (LIG1), and Jun-D increased after inhibiting SUMOylation, suggesting that this mode of post-translational modification regulates CK2 activity [58] (Figure 1).

Altogether, CK2 is sensitive to numerous post-translational modifications that can regulate its activity. 

### 3.2. Protein–Protein Interactions

#### 3.2.1. Activating

Protein–protein interactions have been shown to regulate many roles of CK2 (Table 2). During cell proliferation, fibroblast growth factor 1 (FGF-1) binds to CK2⍺ and CK2β, stimulating the autophosphorylation of CK2β. This may occur through changing the accessibility of the autophosphorylation sites to catalytic CK2 subunits [59]. Similarly, fibroblast growth factor 2 (FGF-2) must bind to nuclear CK2β for the CK2 holoenzyme to phosphorylate nucleolin [60]. Negative elongation factor E (NELFE) also regulates CK2 during cell proliferation. Specifically, increased NELFE expression activates the Wnt/β-catenin pathway through increasing the expression of β-catenin, leading to interactions between β-catenin and the transcription factors TCF/LEF, resulting in increased CK2β expression. This pathway is relevant in gastric cancer proliferation in metastasis [61]. Third, it has been reported that dual specificity mitogen activated protein kinase (MEK) regulates the role of CK2 in promoting the nuclear translocation of ERK within the MAPK pathway. Specifically, the nuclear translocation signal on ERK is occluded, which prohibits phosphorylation by CK2 until stimulation by MEK, which releases ERK, enabling phosphorylation by CK2 and subsequent translocation into the nucleus via importin-7 [62]. The role of CK2 in controlling the activity of krueppel-like factor 4 (KLF4)—a transcription factor implicated in bladder cancer proliferation—is regulated by tumour suppressor p21. This protein interacts with CK2, and increases CK2 phosphorylation of HDAC2 to prevent deacetylation of KLF4, resulting in decreased proliferative activity [63,64].

Protein–protein interactions also regulate the roles of CK2 in cell morphology and cytoskeletal organization. For example, the scaffold protein casein kinase 2-interacting protein 1 (CKIP-1) binds with CK2⍺ (but not CK2⍺′) at the plasma membrane. Localization of CK2, CKIP-1 and PAK1 to the membrane has been reported to allow phosphorylation and activation of PAK1 [70,71,72,73]. 

CK2 is an essential regulator of cell cycle progression, and there are instances of regulation via protein–protein interactions. For example, CK2 has been shown to increase the stability of the tumour suppressor p53, which promotes cell cycle arrest following DNA damage after UV irradiation. Here, CK2 must first associate with the FACT complex (hSpt16 and SSRP1) before p53 phosphorylation can occur [74]. Furthermore, as mentioned earlier, CK2⍺ is temporally phosphorylated by CDK1 during the cell cycle, where phospho-mimetic and -ablative mutations result in defective mitosis [50]. The peptidyl-prolyl cis-trans isomerase Pin1 (Pin1) also interacts with phosphorylated CK2 and has been implicated in modulating the dephosphorylation of CK2 and its localization to the mitotic spindle [67].

Protein–protein interactions have also been shown to regulate the neurological functions of CK2. Studies have shown that following activation of the extracellular domain of L1 cell adhesion molecule (L1CAM) during neuritogenesis, its intracellular domain binds CK2⍺, resulting in phosphorylation of CK2⍺ at T360 and S362 by an unknown kinase and CK2-mediated inhibition of the tumour suppressors phosphatase and tensen homolog (PTEN) and p53 as well as increased neuronal growth [77]. CK2 is also involved in neuronal glutamate signalling through the regulation of N-methyl-D-aspartic acid (NMDA) receptor composition. Increased synaptic activity increases calcium concentrations, leading to activation of Ca^2+^/calmodulin-dependent protein kinase 2 (CaMKII). Then, activated CaMKII creates a complex with CK2 and glutamate receptor ionotropic, NMDA 2B (GluN2B), resulting in CK2-mediated phosphorylation of GluN2B, disrupting its interaction with scaffolding membrane-associated guanylate kinase (MAGUK) proteins, leading to its subsequent shutting away from synapses [82].

Outside of its functions in specific biological pathways, CK2 has been associated with multifaceted stress-response signalling pathways regulated by the chaperone proteins heat shock protein 90 (HSP90) and p38. During heat shock, HSP90 increases CK2 activity by binding CK2⍺ and protecting it from aggregation and inactivation through the formation of stable complexes. Treatment with heparin inhibits this interaction, suggesting that the HSP90:CK2⍺ interaction may occur at the heparin-binding sites on CK2⍺ [75]. A potentially more insidious case of CK2 regulation in response to cellular stress involves the disease acute myeloid leukemia (AML). Healthy cellular signalling relies on maintaining cellular homeostasis through a balance between phosphorylation and dephosphorylation—something that is commonly disrupted in AML. Specifically, p38β overexpression (a hallmark of AML) activates CK2, leading to phosphorylation and cytosolic sequestering of SET, where protein phosphatase 2 (PP2A) is then inactivated, contributing to the loss of homeostasis [66].

A component of healthy blood flow in humans involves regulating blood pressure through the renin–angiotensin–aldosterone system (RAAS). Here, the liver produces the precursor protein angiotensinogen and is cleaved by renin to produce angiotensin I (AT1). AT1 is then converted into angiotensin II (AT2) by angiotensin-converting enzymes, which, through multiple pathways, causes vasoconstriction, increased blood volume, and sympathetic nervous system activation [83]. Small molecules frequently inhibit multiple steps within RAAS to lower patient blood pressure. CK2 is also involved in RAAS by decreasing calcium influx into the heart by inhibiting the Ca^2+^ channel Ca_v_1.2 via phosphorylation at T1704. AT2 controls CK2 here. Specifically, the protein binds to the AT1 receptor which then interacts with β-arrestin 2, which stimulates the kinase SFK, leading to phosphorylation of T88 on p27, preventing it from inhibiting CK2 phosphorylation of Ca_v_1.2 [84].

T cells are an integral component of adaptive immunity, and their production is dependent on the activation of ERK in a CK2-dependent manner. However, CK2 requires activation by CD5 before this can occur. Studies have shown that CD5-increased CK2 activity is 9-fold higher than its activity without any CD5 interaction [69]. Activated CK2 has also been shown to be essential for the generation and differentiation of Th17 cells through glycogen synthase kinase 3 (GSK-3) inhibition and molecular target of rapamycin (mTOR) activation [85].

While hemoglobin is essential for oxygen transport in mammals, it can also contribute to disease. For example, during hemolytic anemia, hemoglobin is released from red blood cells and can cause oxidative stress. However, to prevent this, free hemoglobin binds to haptoglobin, allowing removal by macrophages after interacting with scavenger receptor cysteine-rich type 1 protein M130 (CD163) [86]. CD163 is also involved in regulating the role of CK2 in glioma stemness by interacting with CK2β, leading to an increase in phosphorylation of AKT, GSK-3β/β-catenin pathway activity, inappropriate cell cycle progression, and glioma proliferation [68]. 

The transcription factor SRY-box transcription factor (SOX) 2 (SOX2) is associated with abnormal cell proliferation in esophageal cancer. Studies have shown that SOX2 increases the expression of the microRNA (miRNA) miR-30e, which decreases the expression of ubiquitin carboxyl-terminal hydrolase 4 (USP4), increasing ubiquitination and proteasomal degradation of mothers against decapentaplegic homolog 4 (SMAD4). As SMAD4 has been demonstrated to inhibit CK2, decreased SMAD4 expression would allow CK2 to activate downstream pathways that increase cell migration and proliferation [65].

#### 3.2.2. Inhibitory

Protein–protein interactions can also inhibit the functions of CK2 in other biological contexts that will be highlighted briefly in the following discussion. While Pin1 helps localize CK2⍺ to the mitotic spindle and protects it from dephosphorylation during the cell cycle [67], it also inhibits CK2 phosphorylation of topoisomerase II⍺ (TOP2A) at T1342 [79]. Furthermore, the colon cancer-associated tumour suppressor adenomatous polyposis cell protein (APC) also negatively regulates CK2 during the cell cycle. The study’s authors observed that the physical interaction between APC and CK2⍺ is the highest in G2/M, diminishing during G1/G2, and absent in G0. During G2/M, when its interaction with APC is the strongest, autophosphorylation of CK2⍺ and CK2β was decreased, resulting in lower CK2 activity [87].

Lamin A, a prominent component of the nuclear architecture, interacts with CK2⍺ and CK2⍺′ and sequesters it to the nuclear lamina. This interaction decreases CK2 activity and induces cellular senescence [78]. 

Within the NF-kB signalling pathway in hepatocellular carcinoma, tumor necrosis factor, alpha induced protein 1 (TNFAIP1) was shown to interact with CK2β, promoting its ubiquitination and degradation, suppressing CK2β-dependent NF-kB activation [81].

The role of CK2 in vascular growth is to regulate phosphorylation of PTEN, indirectly controlling AKT1 expression and cell proliferation. However, SMAD4 inhibits CK2 to maintain homeostasis and prevent aberrant growth. Here, the TGFβ ligands bone morphogenetic protein 9/10 (BMP9/10) stimulate activin receptor-like kinase 1 (ALK1) and SMAD4, resulting in decreased CK2 expression and thus inhibiting CK2-mediated inactivation of PTEN. SMAD4 regulation of CK2 has clinical relevance as patients with hereditary hemorrhagic telangiectasia (HHT) suffer from arteriovenous malformations, which have been linked to decreased SMAD4 as this causes unregulated CK2 phosphorylation of PTEN, which is then unable to repress phosphoinositide 3-kinase (PI3K)/AKT signaling [80]. 

During adipogenesis, kinesin family member 5C (KIF5C), responsible for transporting cargo along microtubules, interacts with CK2⍺′, inhibiting its nuclear translocation and kinase activity required for mitotic clonal expansion [76]. 

Overall, these examples illustrate how CK2 relies on other proteins to prevent aberrant phosphorylation by regulating its accessibility to substrates until it is biologically appropriate.

### 3.3. Regulatory Interactions with other Biological Molecules

While protein–protein interactions encompass a large proportion of mechanisms that regulate CK2, there are multiple examples where small, endogenously produced molecules have been reported to regulate the ability of the kinase to phosphorylate its substrates.

#### 3.3.1. Activating

Polyamines—small, endogenously produced, basic organic compounds containing multiple amino groups separated by carbon chains—stimulate CK2 activity. Several in vitro studies have characterized this interaction between CK2 and polyamines. Spermine, the polyamine with the greatest ability to increase CK2 activity [88], binds to T72 on CK2β, near a glutamic acid tripeptide at residues 60, 61, and 63 that is proximal to the catalytic pocket of the kinase [89]. The prevailing theory is that these glutamic acids interact with positive residues within catalytic pockets unbound to polyamines, hindering substrates from interacting with the kinase. Upon polyamine binding, amino groups from the polyamine compete for binding to the acidic glutamic acid residues, inducing a conformation change where the polyamine-binding region is moved out of the catalytic pocket, allowing substrates to be phosphorylated [90]. Notably, overexpression of ornithine decarboxylase 1—the enzyme responsible for catalyzing the first step in synthesizing the polyamines spermine, spermidine, and putrescine—results in increased CK2⍺ and CK2β expression and activity. Interestingly, nuclear CK2 appears unaffected by polyamines, possibly due to competition with macromolecules that may preferentially bind to CK2 at a region that occludes the polyamine binding domain [91].

In pancreatic cells, the transcription factor PDX-1 has a significant role in insulin expression. CK2 is involved in a feedback loop that regulates insulin production by phosphorylating PDX-1 at T231 and S232 [92]. High extracellular insulin concentrations have been reported to cause activation of CK2 and its subsequent phosphorylation of PDX-1 [93]. This phosphorylation leads to an interaction between PDX-1 and the E3 ubiquitin ligase adapter protein phosphorylated CTD interacting factor 1 (PCIF1), targeting it for proteasomal degradation, decreasing insulin expression [94].

Intrauterine growth restriction (IUGR) leads to insulin-like growth factor 1 receptor (IGF-1R)-mediated fetal growth retardation through impaired oxygen and nutrient delivery. In IUGR, hypoxia or leucine deprivation activates CK2 through upregulated mTOR and general control nonderepressible 2 (GCN2) signalling, respectively, which leads to IGF-1R hyperphosphorylation and IGF-1R inhibition [95].

One cellular priority following DNA damage is cell cycle arrest, which commonly occurs via phosphorylation of the tumour suppressor p53 [96]. After DNA damage, inositol hexakisphosphate kinase 2 (IP6K2) catalyzes the production of the inositol pyrophosphate 7 (IP7). IP7 then binds to CK2, which promotes phosphorylation of Tti1 and Tel2, leading to ATM and DNA-dependent protein kinases (DNA-PKcs) stabilization, culminating in p53 phosphorylation at S15, activating downstream cell death pathways [97].

#### 3.3.2. Inhibitory

Bioinformatic analysis of published microarrays revealed that the miRNA miR-1184 has three-fold lower expression in colon cancer tissues than in healthy cells. Subsequent evaluation revealed that CK2⍺ is a principal target of miR-1184. As CK2 has been associated with numerous proliferative and anti-apoptotic functions, it is unsurprising that increased CK2 expression resulting from reduced miR-1184-mediated inhibition is a frequent phenotype in colon cancer [98].

Another potential regulatory interaction that decreases CK2 activity involves the negatively charged polysaccharide heparin, although the physiological relevance of CK2 inhibition by heparin remains to be established. Produced by mammalian mast cells, heparin inhibits blood clotting by interrupting the coagulation cascade through interactions with antithrombin III and is widely used in clinical settings as an anticoagulant. However, heparin also binds to some proteins that are not involved in blood clotting [99], including CK2. Here, heparin electrostatically interacts with the basic K74-KKKI-K79 region on CK2⍺, leading to CK2 inhibition [100].

### 3.4. Hierarchical Phosphorylation

An interesting phenomenon that occurs with acidophilic kinases like CK2 is hierarchical phosphorylation. With hierarchical phosphorylation, when a kinase phosphorylates a residue, the addition of the negative charge primes the kinase to phosphorylate a nearby residue that now conforms to the acidic kinase consensus motif. 

Published examples of hierarchical phosphorylation regulating CK2 activity include the circadian rhythm protein Timeless. Phosphorylation of the protein at residues 297 and 301 by GSK-3 promotes CK2 phosphorylation at residues 305, 309, and 313 [101]. Another instance of regulating CK2 in this manner involves the epithelial ion/water transport protein cystic fibrosis transmembrane conductance regulator (CFTR). Here, CK2 phosphorylation of CFTR S511 requires phosphorylation of Y512 by the tyrosine kinase Fyn. Interestingly, studies have suggested that this occurs more frequently in CFTRΔ508, the variant that causes cystic fibrosis [102,103]. Lastly, hierarchical phosphorylation regulates CK2 phosphorylation of insulin receptor (INSR). Specifically, CK2 phosphorylation of INSR T1160 is dependent on autophosphorylation of residues Y1158, Y1162, and Y1163 [104].

## 4. Unknown Regulatory Mechanism

With the emergence and increased availability of high throughput phosphoproteomics studies, there is an increasing prevalence of studies that reveal phosphorylation of residues within peptides conforming to the CK2 consensus motif. As CK2 was not a focus of these studies, follow-up studies probing the relationship between protein and CK2 were typically not performed. As such, the mechanism of modulating CK2 activity is unknown, but still warrants inclusion here.

### 4.1. Activating

Excitotoxic cell death after neural glutamate overstimulation activates several kinases including CK2 [105]. Although the exact mechanism of this activation is unknown, it may be related to the aforementioned protein–protein interaction where glutamate signalling activates CaMKII, which in turn activates CK2 [82].

It has also been reported that CK2 is regulated by SOX4 within the β-catenin/Wnt signalling pathway. Here, SW480 cells were transfected with FLAG-tagged SOX4, then 24 h later, cells were treated with the CK2 inhibitor TBB. Subsequent Western blotting analysis revealed that β-catenin phosphorylation increased after SOX4 transfection but decreased after treatment with TBB. This suggests that SOX4 regulates CK2-mediated phosphorylation of β-catenin [106].

Interestingly, oncogenic RAS mutations including Q61 and G12 mutations have also been shown to increase CK2⍺ expression and activity. More specifically, one study used stable isotope labeling by amino acids in cell culture (SILAC) to compare the phosphoproteomes of NRAS mutants expressed in primary human melanocytes. Motif analyses revealed that NRAS(Q61L) expression leads to increased phosphorylation of CK2⍺ and CK2⍺′ substrates [107]. Another study used immunofluorescence to demonstrate that HRAS(G12V) targets CK2⍺ to the perinuclear space through the scaffold protein kinase suppressor of Ras 1 (KSR1), where it then phosphorylates CCAAT-enhancer binding protein beta (C/EBPβ) at S222 [108].

### 4.2. Inhibitory

Studies have shown that photobiomodulation (using low-powered light to irradiate tissues) to induce biological effects can treat various diseases, including diabetes and various neural disorders such as Parkinson’s and severe depression [109]. Additionally, blue light photobiomodulation has been shown to inhibit fibroblast activity and may have utility in treating fibrosis. When performing phosphoproteomics on mouse fibroblast cultures after blue light irradiation, researchers identified decreased phosphorylation of phosphosites that conform to the CK2 consensus motif, suggesting that blue light photobiomodulation inhibits CK2 activity [110].

CK2-dependent phosphorylation in liver cells decreased in vivo when mice developed toxicant-associated steatohepatitis (TASH) after introducing the disease with polychlorinated-biphenyl exposure or a high-fat diet. The authors determined that the decreased CK2-dependent phosphorylation was due to attenuated expression of CK2⍺ and CK2β, which resulted in lower caspase-3 phosphorylation and thus increased cell death—which is a hallmark of TASH. Interestingly, however, expression of CK2⍺′ increased. The authors speculated that the latter observation is related to the involvement of CK2⍺′ in interleukin-6 (IL-6) and TNF inflammatory signalling, which increases in TASH [111].

Lastly, a study that examined the role of the nongenotoxic carcinogen pregnenolone carbonitrile (PCN) in mice liver malignancy identified a novel mechanism of inhibiting CK2. PCN binds to the pregnane X receptor (PXR), which, when activated, has been associated with liver enlargement and carcinogenesis. Here, mice were fed PCN for seven days, followed by metabolomic, transcriptomic, proteomic, and phosphoproteomic analyses. The researchers discovered that 9 out of the 21 phosphosites with significantly decreased phosphorylation were CK2 substrates. Gene ontology analysis of seven of these substrates, Hsp90ab1, hepatoma-derived growth factor (HDGF), MTDH, eukaryotic initiation factor 5 beta (EIF5β), zinc finger Ran-binding domain-containing protein 2 (ZRANB2), calnexin, and nuclear ubiquitous casein and cyclin-dependent kinase substrate 1 (NUCKS1), highlighted involved in poly(A) RNA binding, suggesting that PCN may cause malignancy partly through CK2-mediated transcriptional and translational dysfunction [112].

## 5. Summary and Perspectives

As far back as the late 1980s, there were a number of biochemical studies that yielded results suggestive of spatiotemporal regulation of CK2 activity. For example, kinase assays performed using a CK2 peptide substrate (RRADD**S**DDDDD) with extracts from synchronized cells showed increased CK2 activity in nuclear, but not whole-cell, extracts [113]. In a similar respect, kinase assays, again using a CK2 substrate peptide (RRREEE**T**EEE), performed with immunoprecipitates of CK2 from synchronized cell lysates followed revealed modulation of CK2 activity after release from G0 arrest. Specifically, CK2 activity increased 2.5-fold after release from G0 before slowly decreasing to G0-levels 12-h after release and then subsequently increasing slightly and plateauing for the remaining 9 h of the study [114]. While intriguing, results from these sorts of in vitro studies do not necessarily correlate with those in cells and further investigations of CK2 activity in living cells are warranted.

Based on the structures of CK2 and observations that CK2 has catalytic activity without any stimuli, it has become generally accepted that CK2 may be constitutively active. At the same time, there is a large body of evidence demonstrating that the phosphorylation of specific CK2 substrates can be regulated within cells. Moreover, there are dozens of studies, many described here, that highlight specific cases where phosphorylation of a substrate by CK2 is regulated via a variety of distinct mechanisms including post-translational modifications, protein–protein interactions, and regulatory interactions. 

**Figure 2:**
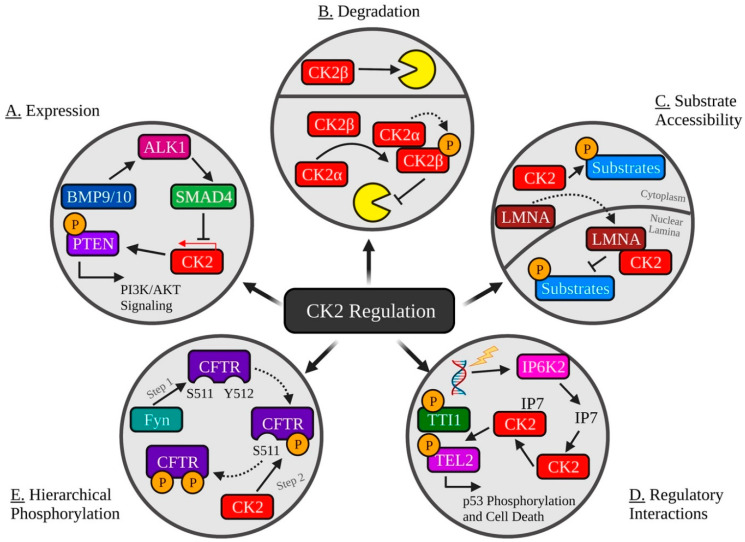
Common Mechanisms of Regulating CK2 Activity. (**A**) The TGFβ ligands BMP9/10 stimulate ALK1 and SMAD4, resulting in decreased CK2 expression and inhibition of CK2-mediated inactivation of PTEN. (**B**) CK2 holoenzyme formation promotes autophosphorylation of CK2β and protection from ubiquitination and degradation. (**C**) Lamin A (LMNA) interacts with CK2⍺ and CK2⍺′ and sequesters it to the nuclear lamina, decreasing CK2 activity. (**D**) DNA damage initiates IP6K2-catalyzed production of IP7, which then binds to CK2, promoting its phosphorylation of Tti1 and Tel2, resulting in p53 phosphorylation and activation of cell death. (**E**) CK2 phosphorylation of CFTR S511 requires phosphorylation of Y512 by the tyrosine kinase Fyn. Created with Biorender.com.

Overall, it is important to emphasize that CK2 is responsible for regulating numerous biological processes that rely on careful control to avoid dysregulation and disease. Through the intrinsic and extrinsic regulatory mechanisms that exist, numerous patterns have emerged (summarized in Figure 2).

Post-translational modifications and small-molecule regulators that directly interact with the kinase accentuate how CK2, like most enzymes, are precisely folded proteins susceptible to changes induced by altered residue polarity. Interestingly, three activity-increasing phosphorylation sites on CK2⍺ are clustered within its activation segment, suggesting that this region may be particularly sensitive to post-translational modifications at least under some circumstances.

Second, several mechanisms that regulate CK2 activity involve protein–protein interactions that regulate substrate accessibility. Proteins can directly interact with CK2, shuttling the kinase to various cellular compartments to promote or prevent substrate phosphorylation. Alternatively, as illustrated by the case of ERK phosphorylation by CK2, there are situations where proteins that do not directly interact with CK2 can regulate its access to a phosphorylation site. 

Third, mechanisms have been described where levels of CK2 can be modulated. Studies have shown that the expression of CK2 subunits can be regulated through elegant transcriptional feedback loops. CK2 levels can also be regulated by the proteasome. For example, it is apparent that autophosphorylation of CK2 can confer protection from ubiquitination and degradation. 

## 6. Concluding Remarks

For the past several decades, considerable attention within the CK2 field has been focused on identifying physiological substrates, its role in the regulation of biological processes, and its involvement in disease. Collectively, these advances have revealed the impact of CK2 as a “master regulator” involved in numerous biological processes both within normal physiological and pathophysiological settings. While it is clear that CK2 is a fascinating kinase with promise as a therapeutic target, there is a notable paradox, pertaining to how CK2 as a constitutively active kinase can be a regulatory participant in critical cellular processes, that remains to be resolved. Deciphering the precise mechanisms that regulate CK2 activity within healthy cells and how these mechanisms are perturbed in pathological processes will both more thoroughly illuminate the impact of CK2 within living systems and may hold promise for capitalizing on CK2 as a therapeutic target for a number of illnesses.

## Figures and Tables

**Table 1:** Alphabetized list of selected CK2 inhibitors.

Inhibitor	Classification	Mechanism	In Clinical Trials?	Refs.
CIGB-300	Peptide	Direct Substrate Binding	Yes	[26]
Emodin	Natural Product	ATP Competitive	Yes ^1^	[27]
KN2	Small Molecule	Bivalent Inhibition	No	[28]
Quinalizarin	Small Molecule	ATP Competitive	No	[29]
SGC-CK2-1	Small Molecule	ATP Competitive	No	[30]
Silmitasertib	Small Molecule	ATP Competitive	Yes	[25]
TBB	Small Molecule	ATP Competitive	No	[31]
TBBz	Small Molecule	ATP Competitive	No	[31]

^1^ Emodin was used in a clinical trial, but the study was prematurely terminated.

**Table 2:** Selected examples of CK2 regulation by extrinsic protein–protein interactions.

Interactor	Effect on CK2 Activity	Affected CK2 Substrate(s)	Biological Function	Refs.
APC	Inhibitory	CK2⍺/CK2β	Cell Proliferation	[65]
AT2	Activating	Ca_v_1.2	Cardiovascular Regulation	[66]
CaMKII	Activating	GluN2B	Glutamate Signalling	[67]
CD163	Activating	AKT	Cell Proliferation	[68]
CD5	Activating	N/A	Adaptive Immunity	[69]
CKIP-1	Activating	PAK1	Cell Morphology	[70,71,72,73]
FACT	Activating	p53	DNA Damage Response	[74]
FGF-1	Activating	CK2β	Cell Proliferation	[59]
FGF-2	Activating	Nucleolin	Cell Proliferation	[60]
HSP90	Activating	N/A	Stress Response	[75]
KIF5C	Inhibitory	N/A	Cell Cycle	[76]
L1CAM	Activating	PTEN; p53	Neuritogenesis	[77]
Lamin A	Inhibitory	N/A	Cellular Senescence	[78]
MEK	Activating	ERK	Cell Proliferation	[62]
NELFE	Activating	N/A	Cell Proliferation	[61]
p21	Activating	HDAC2	Cell Proliferation	[63,64]
p38β	Activating	SET	Stress Response	[66]
Pin1	Activating	N/A	Cell Cycle	[67]
Pin1	Inhibitory	TOP2A	Cell Cycle	[79]
SMAD4	Inhibitory	PTEN	Cell Proliferation	[80]
SOX2	Activating	N/A	Cell Proliferation	[65]
TNFAIP1	Inhibitory	N/A	Cell Proliferation	[81]
